# Consequences of screening in colorectal cancer (COS-CRC): development and dimensionality of a questionnaire

**DOI:** 10.1186/s40359-020-00504-3

**Published:** 2021-01-07

**Authors:** Jessica Malmqvist, Volkert Siersma, Christine Winther Bang, John Brodersen

**Affiliations:** 1 The Research Unit for General Practice and Section of General Practice, Department of Public Health, University of Copenhagen, Oester Farimagsgade 5, 1514 Copenhagen, Denmark; 2 Primary Healthcare Research Unit, Region Zealand, Alléen 15, 4180 Soroe, Denmark

**Keywords:** Patient reported outcome measures, Colorectal neoplasms, Early detection of cancer, Mass screening

## Abstract

**Background:**

Harms of colorectal cancer (CRC) screening include psychosocial consequences. We have not identified studies using a participant-relevant questionnaire with adequate measurement properties to investigate these harms. However, Brodersen et al. have previously developed a core questionnaire consequences of screening (COS) for use in screening for life-threatening diseases. Therefore, the objectives were: (1) To investigate content validity of COS in a CRC screening setting and in case of gaps in content coverage (2) generate new items and themes and (3) test the possibly extended version of COS for dimensionality and differential item functioning (DIF) using Rasch Models.

**Methods:**

We performed two-part-focus-groups with CRC screenees. Screenees were recruited by strategic sampling. In the first part 16 screenees with false-positive results (n = 7) and low-risk polyps (n = 9) were interviewed about their CRC screening experiences and in the second part COS was examined for content validity. When new information was developed in the focus groups, new items covering this topic were generated. Subsequently, new items were, together with COS, tested in the subsequent interviews. A random subsample (n = 410) from a longitudinal questionnaire study, not yet published, was used to form the data for this paper. We analysed multidimensionality and uniform DIF with Andersen’s conditional likelihood ratio test. We assessed individual item fit to the model. We also analysed Local Dependence (LD) and DIF by partial gamma coefficients using Rasch Models.

**Results:**

COS was found relevant in a CRC screening setting. However, new information was discovered in the focus groups, covered by 18 new CRC screening-specific items. The Rasch analyses only revealed minor problems in the COS-scales. The 18 new items were distributed on four new CRC screening-specific dimensions and one single item.

**Conclusion:**

An extended version of COS specifically for use in a CRC screening setting has been developed. The extended part encompasses four new scales and one new single item. The original COS with the CRC-screening specific extension is called consequences of screening in colorectal cancer (COS-CRC). COS-CRC possessed reliability, unidimensionality and invariant measurement.

## Background

Colorectal cancer (CRC) is the third most common type of cancer world-wide [1]. Most cases of CRC are incidental, and even though there are some risk factors of CRC, individual-based interventions on these risk factors are difficult to implement [2, 3]. Therefore, many countries have implemented national screening services for CRC with different modalities such as immunochemical faecal occult blood test (iFOBT), sigmoidoscopy or colonoscopy [4]. In 2014, CRC screening with iFOBT was implemented in Denmark, targeting all individuals aged 50–74 years [5, 6]. All participants with a positive test are urged to undergo a follow-up procedure, which includes bowel preparation and an investigative colonoscopy under local anaesthesia. Besides the intended benefits of early detection, there are potential unintended harms of screening, less frequently reported in the literature [7]. These harms include negative psychosocial consequences, particularly from false-positive results and (over)diagnosis [8–10].

Previous cancer screening research in breast, lung and cervical cancer has revealed different degrees of psychosocial consequences from participating in cancer screening and particularly from receiving a false-positive result [11–15]. However, the outcome measures and study design of cancer screening studies on psychosocial consequences are in general inadequate [16, 17]. Therefore, research using questionnaires with high content validity, sound psychometric properties and study designs including baseline measurements as well as timely assessments are highly needed [18].

Brodersen et al. have previously developed condition-specific questionnaires with high content validity and sound psychometric properties to measure psychosocial consequences of screening for specific cancers and other life-threatening diseases [19–22]. Furthermore, Brodersen et al. found that a common core questionnaire, consequences of screening (COS), was relevant in all these screening settings.

We have not identified studies investigating psychosocial consequences of screening for CRC using a condition-specific questionnaire with high content validity and adequate measurement properties. Furthermore, it has not been investigated whether COS is relevant for use in a CRC screening setting. Therefore, the aims of this study were:

1. To investigate content relevance and content coverage of COS in a CRC screening setting.
2. To generate items and themes relevant in a CRC screening setting, in case of gaps in content coverage in the present COS.
3. To test the possibly extended version of COS for dimensionality and differential item functioning (DIF) using Item Response Theory Rasch Models.

## Methods

### The COS questionnaire

The COS questionnaire was originally developed in a breast cancer screening setting [19, 23]. Subsequently, a core-set of nine dimensions and one single item from this first COS questionnaire has been confirmed relevant and has been statistically validated in various other screening settings [20–22].

**Fig. 1:**
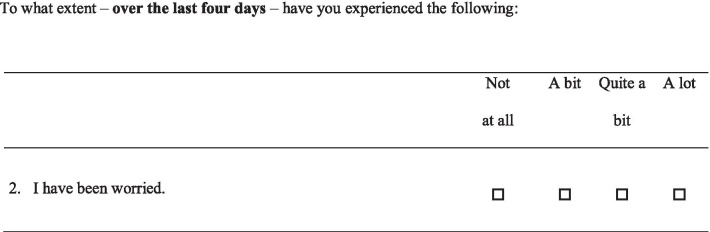
Response categories, COS part I

**Fig. 2:**
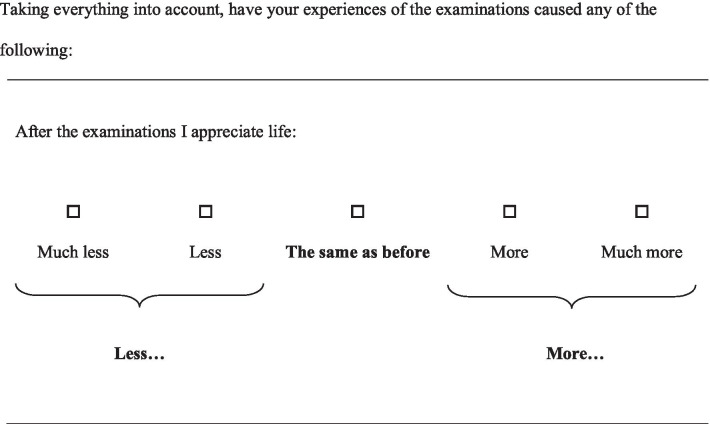
Response categories, COS part II

The core-COS consists of two parts: part I, encompassing four dimensions and one single item, which is relevant before, at, and after screening and for control persons not invited to screening, and part II, encompassing five dimensions, which is only relevant when a screened participant has received a final diagnosis (Table 1). In part I, all items are phrased as the example in Fig. 1*,* with a common stem as in Fig. 1 as a heading of every five to six items. In part II, the items are phrased as in Fig. 2, with a common stem as in the Fig. 2 as a heading of each page in the questionnaire.

**Table 1:** Content of the core-questionnaire COS (consequences of screening)

Themes or single items	The items of COS. The number indicates the order of appearance in the questionnaire
*Part I*	
Anxiety	2. Worried about my future
	3. Scared
	12. Upset
	13. Restless
	14. Nervous
	23. Terrified
	46. Shocked
Behavioural	4. Irritable
	5. Quieter than normal
	8. Hard to concentrate
	10. Change in appetite
	17. Withdrawn into myself
	20. Difficulty dealing work or other commitments
	22. Difficulty doing things around the house
Sense of dejection	1. Worried
	9. Time passed slowly
	11. Sad
	15. Uneasy
	18. Unable to cope
	19. Depressed
Sleep	6. Slept badly
	16. Taken long time to fall asleep
	21. Woken up far too early in the morning
	24. Awake most of the night
Single item	7. Busy to take mind off things
*Part II*	
Relaxed/calm	3. Relaxed
	7. Calm
	15. Relieved
Social relations	4. Family
	5. Friends
	6. Other people
Existential values	1. Broader aspects of life
	2. Enjoyment of life
	8. Thought about future
	9. Well-being
	10. Awareness of life
	11. Value life
Impulsivity	12. Energy
	14. Lived life to the full
	17. Being impulsive
	19. Desire to venture into something new
	20. Desire to venture into something risky
	21. Done some things that overstepped one’s bounds
Empathy	13. Responsibility for one’s family
	16. Understand other people’s problems
	18. Ability to listen to other people’s problems

Furthermore, in the construction of the COS questionnaires for the various other screening settings additional condition-specific dimensions have been developed and validated for use in these specific screening settings [20–22]. Five of these condition-specific dimensions (‘Introvert’, ‘Change in body perception’, Fear and powerlessness’, ‘Change in perception of own age’, and ‘Emotional reactions’) were assumed relevant before at and after CRC screening as well. Hence, they were added as domains to part I of the COS questionnaire for CRC screening that was to be developed.

In core-COS part I, the response options are arranged in four categories from ´Not at all´ to ´A lot´ (Fig. 1). The response scores range from 0 to 3, where 0 corresponds to ‘Not at all’ and 3 to ‘A lot’.

In core-COS part II, the response options are arranged in five categories with `No change´ placed in the middle and two response categories on each side indicating change in opposing directions (less/more change) (Fig. 2). The response category scores range from 0–2 in both directions, where 2 indicates most change.

### Design and setting

This study consisted of two phases: (1) a qualitative phase where content relevance and content coverage of COS in a CRC screening setting were investigated and new items were generated in case of gaps in content coverage (2) a quantitative phase where the possibly extended version of COS was tested for unidimensionality and DIF.

### Phase 1, Qualitative phase

The qualitative phase of this study was conducted as an independent, but connected, part of an explorative qualitative study using focus groups to investigate experiences of receiving a false-positive CRC screening result [24]. The rationale for only including participants with false-positive results and low-risk polyps in the focus groups was based on research in mammography screening, where participants with false-positive results experience the most psychosocial consequences [25, 26]. This group, together with the low-risk polyp group were thus the most relevant groups to uncover the psychosocial consequences of CRC screening. The group with normal results has in previous been shown to be least affected psychosocially, why this group would not contribute with new information, not already revealed by informants with polyps or a false-positive result [26].

The explorative qualitative study including details on recruitment, sampling, and participant characteristics has been published elsewhere [24]. Here we describe how the data was used in this validation study and the additional data collection and analysis.

Four focus groups were performed in Region Zealand, Denmark in 2015 with 16 participants in total. The first interview included five women diagnosed with low-risk polyps, the second included three men with a false-positive result, the third included four women with a false-positive result and the final interview included four men diagnosed with low-risk polyps.

The focus groups were divided into two parts: (a) an explorative part, and (b) a structured part focused on the development and content validation of the questionnaire. The two parts were held in continuation of each other with a break between the explorative part and the structured part. In the first part we explored the experiences of receiving a false-positive CRC screening result by open-ended discussions and in the second part we specifically investigated content relevance and content coverage of the core-COS together with the condition-specific dimensions. Moreover, all items were tested for understandability and ease of completion. We also introduced different phrasing of the items to the participants, to find the most appropriate alternatives.

Each focus group was audio-recorded and lasted 55–90 min.

During the focus groups, JB together with the co-authors of the explorative study acted as moderators and JM as an observer.

### Development and test of new items

In cases where topics not covered in the existing COS were discussed in the explorative part of the focus groups, new items covering these topics were developed. This was partly performed during the focus groups together with the participants and partly by JB in-between focus groups based on the analysed transcripts. When new items had been developed, these were integrated with the existing items to a new draft questionnaire, which was tested in the following focus group. Hence, the test of COS and the development of new items was an iterative process that was performed continuously throughout the data collection process.

Investigation of content validity of new items was performed alongside with the investigation of content validity of COS.

### Data analysis

The focus groups were audio-recorded and transcribed verbatim and a systematic text condensation approach was used to analyse data from the explorative part of the focus groups [27]. Data were analysed in between each focus group and findings used within the next focus group.

### Single interviews

The final draft version of the questionnaire was further tested for understandability and functionality by “*think-aloud-test*” in five single interviews after the four group interviews. The sample was found using convenience sampling of individuals in the target population of the CRC screening programme. In the single interviews JM acted as a moderator. In case of any problems with understandability or comments on functionality, these were discussed between JM and JB and decisions on possible changes were made by these two authors.

### Recall period

The recall period is the period back in time that the respondents are supposed to refer to when responding to the questionnaire. The choice of length of the recall period depends on the outcome to be measured and the design and setting of the study.

The recall period of this questionnaire was discussed and decided within the author group. Hence, neither focus group nor single interview participants were involved in this decision.

### Phase 2, Quantitative phase

#### Data collection for statistical psychometric properties analyses

The data used for the statistical assessment of the psychometric properties was a subset of data collected for a longitudinal questionnaire study, not published yet, aiming to quantify the psychosocial consequences of CRC screening.

In the questionnaire study, the questionnaire was sent to all positive screenees and to age-, sex-, and municipality-matched negative screenees, non-attendees and control persons in a 2:1 design. In total we sent the questionnaire to 4178 individuals eight weeks after their matched positive screenees had received their final diagnosis. The final diagnoses were: CRC, medium- and high-risk polyps, low-risk polyps and clean colon. Hence, positive screenees could be classified in these four categories.

#### Sample size

There is no consensus on an appropriate sample size in Item Response Theory using Rasch Models. Nevertheless, the COSMIN Risk of bias checklist refers to a sample size of ≥ 200 subjects as ‘very good’ [28]. Moreover, previous experiences with Rasch analyses have shown that with samples of 1000 subjects all results tend to be rejected (type I error) i.e. no scales would seem to have adequate fit to the model due to too large power of the study [29]. Therefore, we assumed that a sample of approximately 400 subjects or approximately 60 subjects in each of the seven subgroups was an appropriate sample size. The CRC group was too small; hence, all 50 respondents were included (Table 2).

**Table 2:** Quantitative phase, participant characteristics

Study group	Control persons (n = 60)	Negative screening result (n = 60)	Non-attendees (n = 60)	False-positive screening result (n = 60)	Low-risk polyps (n = 60)	Medium and high-risk polyps (n = 60)	Colorectal cancer (n = 50)
Sex							
Male	33	39	23	28	34	41	31
Female	27	21	37	29	24	19	19
Age							
< 61	18	10	11	18	13	14	7
61–65	10	11	16	6	15	15	8
66–70	22	21	20	17	16	19	14
> 70	10	18	13	16	14	12	21

#### Statistical analyses of dimensionality

The analytical approach was to see whether the data fitted a Rasch model so the investigated scale possessed all the advantageous psychometric properties inherent to the Rasch model [30]. When the items fit the Rasch model the patient-reported outcome measure possesses criterion-related construct validity and is proved to be objective, sufficient, and reliable [23].

Firstly, we investigated unidimensionality, then we investigated absence of differential item functioning (DIF) and lastly, we investigated local independence [30, 31]. When these criteria were fulfilled, the scales and items fit the Rasch model.

Unidimensionality is the ability of a scale only to measure one aspect of a latent trait.

Differential item functioning (DIF), is when an item is excessively correlated to an exogenous variable, and therefore, functions differently in different group of respondents. DIF can be further divided into uniform, when the DIF is constant across the latent trait, and non-uniform DIF, when the DIF vary across the latent trait. Uniform DIF can be adjusted for, while non-uniform DIF cannot [32]. Local independence is when responses to an item are conditionally independent, meaning that two items in a scale only correlate because they both measure the same latent trait.

For each domain, we analysed unidimensionality and uniform DIF with Andersen’s conditional likelihood ratio test (CLR-χ^2^) [33]. Then individual item fit to the partial credit Rasch model for polytomous items was assessed by conditional infits and outfits and by comparing observed and expected item responses for individuals as well as for study groups [34]. Finally, we analysed uniform DIF for subgroups and LD for particular items by partial gamma coefficients using graphical loglinear Rasch Models [35].

The items were assessed by covariates for DIF. The covariates were age, sex and screening result, which have previously been proven relevant in screening settings [22].

When an item or set of items did not fit the model, we analysed data to locate the source of misfit. Furthermore, we re-read the phrasing of all items in that domain to locate any linguistically poorly defined items or any distinction in the meaning of the items indicating that the item belonged to another domain than we had initially hypothesised.

When an item possessed DIF, the scale was analysed both without that item and with split for that item regarding the covariate of which the item possessed DIF. If DIF was uniform and hereby corrected by the split, then the overall fit to the model would increase.

We decided to keep any item in the model as far as the item did not have non-uniform DIF or low content validity.

The Benjamini–Hochberg procedure was used to correct for multiple testing and Cronbach’s alpha was used to assess reliability [36, 37].

We used DIGRAM to perform all the statistical analyses [38].

## Results

### Phase I, qualitative phase

The items in core-COS as well as the items in the condition-specific domains were all found relevant by the participants. Moreover, participants found all items understandable and easy to complete.

The first part of the interviews generated new information on experiences of the CRC screening. Uncomfortableness, pain, perceived burden of drinking the laxative and being bound to one´s home during the bowel preparation were CRC screening-specific topics discussed during the explorative part of the focus groups, that were not covered in the previous version of COS. Embarrassment, pain, and vulnerability related to the colonoscopy as well as uncertainty of the screening result and opinions on participation were other new topics not covered in the previous COS.

These topics were covered in a total of 18 newly developed items that were divided into three new a priori domains: ‘Perceived burden of bowel preparation’, ‘Negative colonoscopy experiences’ and ‘Knowledge of having colorectal polyps’. These new items were all found relevant by participants in the subsequent interviews. The wording of the items was also found understandable and there were no difficulties in completing the items.

These extra a priori domains formed a new part of the questionnaire, specifically for use in CRC screening ‘part Ix ‘. The new domains were only relevant after screening and only to participants who had undergone a colonoscopy following a positive screening result. Hence, these domains naturally fall outside COS part I and II. We assumed that the items and response categories in these a priori domains would have the same structure as the items in part I of the COS questionnaire.

Finally, an item originally developed for lung cancer screening, now modified to fit a CRC screening setting, was found relevant among the interviewees. We assumed that this item ‘Fear of CRC has, more than usual, been in the back of my mind’ would fit in the COS scale ‘Introvert’.

The 18 new items were all developed during the first two focus groups. After the second focus group no new information was discovered. Hence, no further items were generated from data collected in these interviews. The final draft questionnaire was therefore tested in its full version in the two final focus groups; one with women (n = 4) and one with men (n = 4) [24].

Two items on worries about CRC and believe in not having CRC, originally developed for breast cancer and belonging to part II of the original COS, were found relevant by the participants. These items were not included in the survey questionnaire, due to personnel error and the validation of the corresponding two-item scale could therefore not be performed in this study.

### Single interviews, think-a-loud-test

Four women and one man were interviewed. The man and one of the women were interviewed on the street outside a shopping mall while three women were University administrative employees, interviewed at work.

One woman was uncertain about the meaning of item 53 ‘Worried about drinking other fluids during emptying of bowel’ in the new a priori domain ‘Perceived burden of bowel preparation’. Since she had not attended the screening programme yet, we assumed that her uncertainty was related to the fact that it was read out of context. No other comments on the phrasing of the items or the content was revealed during the interviews.

### Recall period

The recall period for the questionnaire was set to four days. The decision about a recall period of four days was a pragmatic decision made by JB and JM. The time window from receiving a positive iFOBT result to undergoing the follow-up colonoscopy can be as narrow as five days or as broad as ten days. To capture the possible psychosocial consequences of being in limbo of having received a positive iFOBT result, waiting for the diagnostic colonoscopy but without being in the middle of emptying of the bowel led us to this decision.

### Phase 2, Quantitative phase

#### Part I

Firstly, we evaluated unidimensionality, then we evaluated absence of DIF and lastly, we evaluated local independence, for each domain.

The four core-COS part I scales ‘Dejection’, ‘Anxiety’, ‘Behaviour’ and ‘Sleep’ exhibited adequate fit of the Rasch model and no items in these scales possessed DIF (Table 3).

**Table 3:** Fit statistics and Cronbach’s alpha of the dimensions of the COS-CRC

Dimensions (number of items)	CLR-χ^2(a)^	Degrees of freedom	P	Cronbach’s alpha
*Part I*				
Sense of dejection (6)	33.1	17	0.011	0.923
Anxiety (7)	16.9	20	0.661	0.920
Behaviour (7)	15.7	20	0.736	0.905
Sleep (4)	15.1	11	0.178	0.894
Introvert (5)	37.5	17	0.003*	0.886
Introvert (4) after deletion of item 26	6.3	14	0.959	0.876
Change in body perception (3)	2.4	8	0.965	0.900
Fear & Powerlessness (4)	5.0	11	0.933	0.892
Change in perception of own age (2)	0.5	5	0.994	0.788
Emotional reactions (3)	10.1	8	0.260	0.797
Emotional reactions (2) after deletion of item 41	0.0	4	1.000	0.873
Emotional reactions with item 41 split for diagnosis	17.1	22	0.757	0.797
Life-style changes (2)	0.4	5	0.996	0.741
Sexuality (2)	31.6	5	0.000*	0.906
*Part Ix*				
Perceived burden of bowel preparation (7)	18.3	20	0.564	0.886
Perceived burden of bowel preparation (6) after deletion of item 53	19.6	17	0.295	0.891
Knowledge of having colorectal polyps (2)	1.8	5	0.879	0.890
Negative colonoscopy experiences (9)	30.6	26	0.245	0.896
Negative physical colonoscopy experiences (3)	3.9	8	0.867	0.892
Negative emotional colonoscopy experiences (5)	13.3	14	0.502	0.918
*Part II*				
Social relations (3)	0.8	5	0.978	0.808
Relaxed/calm (3)	0.0	5	1.000	0.733
Impulsivity (6)	5.0	11	0.934	0.887
Existential values (6)	6.4	11	0.846	0.863
Existential values (5) after deletion of item 11	18.3	9	0.032	0.851
Existential values (6) with item 11 split for age	11.0	15	0.750	0.863
Existential values (6) super item 10 and 11	7.5	14	0.914	0.863
Empathy (3)	12.4	5	0.029	0.833

^a^CLR-χ^2^: Andersen’s conditional likelihood ratio test

^*^Benjamini–Hochberg rejects all p-values less than 0.0037 to control the FDR at 0.05

We found LD in three pairs of items in the ‘Dejection’-scale: item 1 and 8, item 8 and 10, and item 10 and 18 (Table 4).

**Table 4:** Results from the psychometric analyses of part I of the COS-CRC

COS-CRC Part I: item order in the questionnaire	Subscales and misfit	Observed	Expected	Gamma sd	Probability of fit to the Rasch model^a^	Item difficulty	Single or ‘poor’ item
1. Worried	Dejection	0.848	0.894	0.018	0.00888	− 0.73	
2. Worried about my future	Anxiety	0.873	0.887	0.019	0.45578	− 0.92	
3. Scared	Anxiety	0.898	0.886	0.021	0.59302	− 0.40	
4. Irritable	Behaviour	0.858	0.850	0.026	0.73171	− 0.10	
5. Quieter than normal	Behaviour	0.838	0.854	0.024	0.51721	− 0.26	
6. Slept badly	Sleep	0.895	0.833	0.025	0.01292	− 0.16	
7. Hard to concentrate	Behaviour	0.907	0.850	0.026	0.02683	− 0.20	
8. Time passed slowly	Dejection	0.845	0.892	0.021	0.02684	0.26	
9. Change in appetite	Behaviour	0.760	0.851	0.030	0.00210*	0.32	
10. Sad	Dejection	0.934	0.890	0.020	0.02943	0.15	
11. Upset	Anxiety	0.914	0.890	0.023	0.28643	0.29	
12. Restless	Anxiety	0.831	0.884	0.022	0.01562	0.20	
13. Nervous	Anxiety	0.918	0.886	0.020	0.11772	− 0.53	
14. Uneasy	Dejection	0.936	0.891	0.019	0.01951	− 0.00	
15. Taken long time to fall asleep	Sleep	0.817	0.830	0.026	0.62877	0.05	
16. Withdrawn into myself	Behaviour	0.893	0.847	0.028	0.09226	0.21	
17. Unable to cope	Dejection	0.902	0.896	0.024	0.80840	0.98	
18. Depressed	Dejection	0.909	0.893	0.021	0.44646	0.35	
19. Difficulty dealing with work or other commitments	Behaviour	0.827	0.854	0.028	0.33385	0.14	
20. Woken up far too early in the morning	Sleep	0.751	0.831	0.025	0.00137*	− 0.28	
21. Difficulty doing things around the house	Behaviour	0.853	0.850	0.028	0.93310	0.17	
22. Terrified	Anxiety	0.915	0.914	0.029	0.96312	1.32	
23. Awake most of the night	Sleep	0.889	0.832	0.029	0.04738	0.73	
24. Felt sorry for myself	Introvert	0.874	0.868	0.028	0.83291	0.68	
25. Shocked	Anxiety	0.915	0.896	0.025	0.45338	0.78	
26. Fear of CRC has, more than usual, been in the back of my mind	Introvert, misfit	0.775	0.836	0.024	0.01016	− 0.72	‘Poor item’
27. Insecure	Introvert	0.875	0.867	0.024	0.71943	0.04	
28. Felt as though something is wrong with my body	Change in body perception	0.918	0.920	0.018	0.89586	− 0.58	
29. Felt as though my body was a machine that does not work	Change in body perception	0.934	0.919	0.020	0.47468	0.84	
30. Thought my situation was hopeless	Introvert	0.951	0.882	0.028	0.01395	0.55	
31. Experienced that I lost control	Fear & Powerlessness	0.939	0.920	0.025	0.44568	0.92	
32. Experienced mood swings	Introvert	0.885	0.868	0.023	0.46397	0.04	
33. Thought my body was vulnerable	Change in body perception	0.917	0.923	0.018	0.73558	0.23	
34. Kept my thoughts to myself	Introvert	0.824	0.877	0.021	0.01050	− 0.81	
35. Felt that I am getting old	Change in perception of own age	0.894	0.894	0.029	0.99083	− 0.38	
36. Felt sour (attitude)	Emotional reactions minus item 41	0.964	0.964	0.013	0.99810	4.08	
36. Felt sour (attitude)	Emotional reactions with split item 41 for diagnosis	0.912	0.880	0.028	0.26157	1.57	
37. Angry	Emotional reactions minus item 41	0.964	0.964	0.013	0.99810	2.23	
37. Angry	Emotional reactions with split item 41 for diagnosis	0.907	0.880	0.028	0.35081	0.21	
38. Felt I have been in a vacuum	Fear & powerlessness	0.916	0.890	0.024	0.29461	− 0.22	
39. Felt older than my age	Change in perception of own age	0.894	0.894	0.029	0.99083	0.58	
40. Felt powerless	Fear & powerlessness	0.885	0.889	0.025	0.85095	0.02	
41. Frightened	Emotional reactions, misfit	0.792	0.876	0.028	0.00262*	− 0.43	‘Poor item’
41. Frightened	Emotional reactions	0.792	0.861	0.030	0.02251	Negative results: 1.57 Control persons: 1.04 Non-attendees: 0.22 Clean colon: − 1.24 Benign polyps: − 0.68 Adenomatous polyps: − 0.47 CRC: − 1.38	
42. Felt I was unlucky	Fear & powerlessness	0.846	0.880	0.025	0.17683	− 0.39	
43. Have changed diet	Lifestyle changes	0.864	0.863	0.031	0.98433	0.08	‘Single item’
44. Have changed exercise habits	Lifestyle changes	0.864	0.863	0.031	0.98433	0.06	‘Poor item’
45. Less interest in sex	Sexuality	0.959	0.959	0.013	0.98287	0.04	‘Poor item’
46. Negative impact on sex life	Sexuality	0.959	0.959	0.013	0.98287	0.41	‘Single item’

^a^Benjamini-Hochberg rejects all p-values < 0.0033 to control the FDR at 0.05 and < 0.0002 to control the FDR at 0.01

Misfit after adjusted for multiple testing by the Benjamini–Hochberg procedure: * < 5% false discovery rate (FDR), ** < 1% FDR

Two pairs of items in the ‘Anxiety’-scale had LD: item 3 and 13, and item 11 and 12.

In the scale ‘Behaviour’ LD appeared in six pairs of items: items 4 and 5, items 4 and 7, items 4 and 16, items 5 and 16, items 5 and 19, and items 19 and 21.

In the ‘Sleep’-scale we found LD in three pairs of items: item 6 and 15, item 15 and 20, and item 20 and 23.

The scale ‘Introvert’ had overall misfit to the model (Table 3). Furthermore, item 26 ‘Fear of CRC has, more than usual, been in the back of my mind’ had DIF related to the exogenous variable ‘Diagnosis’. Since this item had neither fitted the model in a lung cancer screening setting, this item was removed from the model. Thereafter, the overall fit increased and no items in the scale possessed DIF. There was LD in six pairs of items: items 24 and 27, items 24 and 30, items 24 and 34, items 27 and 34, items 30 and 32, and items 32 and 34.

The three scales: ‘Change in body perception’, ‘Fear and powerlessness’-scale and ‘Change in perception of own age’ all had an overall good fit to the model. None of the items in these three scales possessed DIF or had LD to each other.

In general, the scale ‘Emotional reactions’ fitted the model adequately. However, item 41 ‘Frightened’ had DIF related to the exogenous variable ‘Diagnosis’. Therefore, we tested the model without this item and with split of the item for the variable ‘Diagnosis’. Splitting item 41 for the variable ‘Diagnosis’ revealed uniform DIF.

After we removed item 41 the scale fitted the model adequately and there were no DIF or LD.

The scale ‘Sex’ had overall misfit to the Rasch model. Furthermore, item 45 ‘Less interest in sex’ had DIF and the pairs of items 45 and 46 had LD. Therefore, item 45 was removed and item 46 was kept as a single item.

The ‘Lifestyle changes’-scale had good overall fit to the model. However, item 44 ‘Change in exercise habits’ had DIF and the two items forming the scale had LD. Therefore, item 44 was removed from the model, and item 43 was kept as a single item (Table 4).

#### Part Ix

The CRC-specific scale ‘Perceived burden of bowel preparation’ had overall good fit to the model (Table 5). The scale had LD for the pairs of items: 47 and 50, 47 and 52, 47 and 53, 48 and 49, 48 and 51, 48 and 53, 49 and 51, 49 and 53, 50 and 53, and 51 and 53. Item 53 ‘Worries about drinking other beverages during the bowel preparation’ had DIF related to the exogenous variable ‘Diagnosis’. Therefore, we tested the model without item 53. After removing item 53, the scale still fitted the model, no items possessed DIF and the pairs of items 48 and 49, 48 and 51, and 49 and 51 had LD.

**Table 5:** Results from the psychometric analyses of part Ix of the COS-CRC

COS-CRC Part Ix: item order in the questionnaire	Subscales and misfit	Observed	Expected	Gamma sd	Probability of fit to the Rasch model^a^	Item difficulty	Single or ‘poor’ item
47. Discomfort with having bowel emptied	Perceived burden of bowel preparation	0.763	0.736	0.040	0.49617	− 1.08	
48. Feeling of having a lesion on rectum	Perceived burden of bowel preparation	0.830	0.775	0.047	0.24357	0.79	
49. Stinging feeling in the rectum	Perceived burden of bowel preparation	0.822	0.759	0.047	0.18284	0.85	
50. Pain during emptying of bowel	Perceived burden of bowel preparation	0.754	0.792	0.047	0.42003	0.86	
51. Feeling of a rash on backside	Perceived burden of bowel preparation	0.805	0.759	0.047	0.33519	0.82	
52. Feeling stranded at home during emptying of bowel	Perceived burden of bowel preparation	0.661	0.737	0.041	0.06132	− 1.98	
53. Worried about drinking other fluids during emptying of bowel	Perceived burden of bowel preparation	0.605	0.736	0.046	0.00444	0.34	‘Poor item’
54. Anxious about having one or more polyps	Knowledge of having colorectal polyps	0.936	0.936	0.029	0.98862	− 2.88	
55. Confused about what it means to have one or more polyps	Knowledge of having colorectal polyps	0.936	0.936	0.029	0.98862	3.00	
56. Discomfort during the colonoscopy	Negative experiences of the colonoscopy	0.742	0.739	0.034	0.91764	− 1.17	
56. Discomfort during the colonoscopy	Negative physical experiences of the colonoscopy	0.900	0.870	0.027	0.26895	− 0.77	
57. Felt defenseless lying on the examination table	Negative experiences of the colonoscopy	0.840	0.735	0.038	0.00567	− 0.23	
57. Felt defenseless lying on the examination table	Negative emotional experiences of the colonoscopy	0.850	0.868	0.025	0.49037	− 0.53	
58. Felt vulnerable lying on the examination table	Negative experiences of the colonoscopy	0.854	0.735	0.038	0.00188*	− 0.19	
58. Felt vulnerable lying on the examination table	Negative emotional experiences of the colonoscopy	0.894	0.871	0.025	0.36890	− 0.46	
59. Pain during the colonoscopy	Negative experiences of the colonoscopy	0.598	0.742	0.035	0.00004**	− 0.67	
59. Pain during the colonoscopy	Negative physical experiences of the colonoscopy	0.855	0.860	0.026	0.85238	0.06	
60. Humiliation lying on the examination table	Negative experiences of the colonoscopy	0.698	0.727	0.043	0.50161	0.45	
60. Humiliation lying on the examination table	Negative emotional experiences of the colonoscopy	0.844	0.856	0.030	0.69602	0.59	
61. The examination was a harsh experience	Negative experiences of the colonoscopy	0.786	0.742	0.040	0.27558	0.01	
61. The examination was a harsh experience	Negative physical experiences of the colonoscopy	0.852	0.871	0.027	0.47659	1.28	
62. Lying on the examination table overstepped my boundaries	Negative experiences of the colonoscopy	0.684	0.725	0.046	0.37364	0.60	
62. Lying on the examination table overstepped my boundaries	Negative emotional experiences of the colonoscopy	0.856	0.854	0.031	0.96742	0.95	
63. Felt exposed lying on the examination table	Negative experiences of the colonoscopy	0.737	0.726	0.046	0.81139	0.58	
63. Felt exposed lying on the examination table	Negative emotional experiences of the colonoscopy	0.878	0.852	0.031	0.40937	0.87	
64. Regrets having participated in the screening programme	Regret participation	N/A	N/A	N/A	N/A	N/A	‘Single item’

^a^Benjamini-Hochberg rejects all p-values < 0.0033 to control the FDR at 0.05 and < 0.0002 to control the FDR at 0.01

^*^ < 5% false discovery rate (FDR), ** < 1% FDR

The scale ‘Knowledge about colorectal polyps’ fitted the Rasch model, the items possessed no DIF or LD.

Our hypothesis of items 56–64 forming the scale ‘Negative colonoscopy experiences’ had overall fit to the model and no items possessed DIF. LD was revealed in 25 pairs of items and several items had misfit to the model. These results could indicate two- or multi-dimensionality. Therefore, we re-read all the items in this scale to reconsider whether there was more than one dimension hidden in this scale. We decided to split the scale into a physical part: item 56, 59 and 61, and a psychological part: item 57, 58, 60, 62, and 63. After re-reading item 64 about post-participation opinion we agreed on keeping it as a single item since it had been declared relevant to the participants and had not possessed DIF in the initial analyses, but linguistically it did not fit into any of the existing scales. The new scale ‘Negative physical colonoscopy experiences’ had overall fit to the model and the items possessed no DIF. One pair of items 56 and 59 had LD.

The scale ‘Negative emotional colonoscopy experiences’ also fitted the model and no items possessed DIF. The pairs of items 57 and 58, 57 and 60, 57 and 62, 58 and 60, and 62 and 63 had LD.

#### Part II

The four COS part II scales ‘Social relations’, ‘Relaxed/calm’, ‘Impulsivity’ and ‘Empathy’ had overall good fit to the Rasch model (Table 6). Furthermore, neither of the items possessed DIF. The ‘Impulsivity’-scale had LD for the pairs of items 16 and 19, 16 and 20, 19 and 20, and 20 and 21. The ‘Empathy’-scale had LD for the pairs of items 4 and 5 and 5 and 15.

**Table 6:** Results from the psychometric analyses of part II of the COS-CRC

COS-CRC Part II: item order in the questionnaire	Subscales and misfit	Observed	Expected	Gamma sd	Probability of fit to the Rasch model^a^	Item difficulty	Single or ‘poor’ item
1. Broader aspects of life	Existential values after deletion of item 11	0.815	0.833	0.033	0.57423	− 0.50	
1. Broader aspects of life	Existential values with item 11 split for age	0.820	0.844	0.030	0.41669	− 0.43	
1. Broader aspects of life	Existential values super item 10 and 11	0.820	0.827	0.033	0.81774	− 0.51	
2. Enjoyment of life	Existential values after deletion of item 11	0.827	0.816	0.038	0.77772	0.15	
2. Enjoyment of life	Existential values with item 11 split for age	0.827	0.833	0.034	0.87726	0.21	
2. Enjoyment of life	Existential values with super item 10 and 11	0.827	0.812	0.037	0.68256	0.07	
3. Relaxed	Relaxed/calm	0.728	0.736	0.050	0.87581	0.05	
4. Ability to listen to other people’s problems	Empathy	0.949	0.933	0.023	0.24502	0.26	
5. Understand other people’s problems	Empathy	0.964	0.937	0.021	0.20138	− 0.37	
6. Family relationships	Social relations	0.972	0.976	0.011	0.69000	− 1.60	
7. Friend relationships	Social relations	0.980	0.977	0.012	0.77011	0.58	
8. Other people relationships	Social relations	0.978	0.967	0.016	0.51007	1.02	
9. Calm	Relaxed/calm	0.758	0.729	0.053	0.59002	0.91	
10. Thoughts about future	Existential values after deletion of item 11	0.741	0.813	0.040	0.07193	0.27	
10. Thoughts about future	Existential values with item 11 split for age	0.821	0.833	0.036	0.73595	0.34	
10. Thoughts about future	Existential values with super item 10 plus 11	0.821	0.872	0.030	0.08849	0.45	
11. Well-being	Existential values, misfit	0.828	0.829	0.038	0.98421	0.52	‘Poor item’
11. Well-being	Existential values with split for age	0.828	0.815	0.040	0.74393	Age = < 61: 14.02 Age = 61–65: − 0.32 Age = 66–70: 14.02 Age = > 70: 0.70	
11. Well-being	Existential values with super item 10 plus 11	0.828	0.874	0.031	0.13929	0.06	
12. Awareness of life	Existential values with deletion of item 11	0.855	0.818	0.038	0.32697	0.23	
12. Awareness of life	Existential values, with item 11 split for age	0.854	0.833	0.034	0.53923	0.29	
12. Awareness of life	Existential values with super item 10 and 11	0.854	0.813	0.037	0.26599	0.14	
13. Value life	Existential values after deletion of item 11	0.868	0.827	0.035	0.24845	− 0.16	
13. Value life	Existential values with item 11 split for age	0.862	0.839	0.032	0.48078	− 0.09	
13. Value life	Existential values with super item 10 and 11	0.862	0.821	0.034	0.23802	− 0.21	
14. Energy	Impulsivity	0.864	0.907	0.030	0.14298	0.31	
15. Feel responsible for my family	Empathy	0.854	0.913	0.025	0.02108	0.11	
16. Lived life to the full	Impulsivity	0.900	0.912	0.030	0.68793	− 0.45	
17. Relieved	Relaxed/calm	0.726	0.753	0.047	0.56101	− 0.96	
18. Being impulsive	Impulsivity	0.913	0.909	0.032	0.91435	0.70	
19. Desire to venture into something new	Impulsivity	0.930	0.906	0.030	0.43608	− 0.26	
20. Courage to venture into something risky	Impulsivity	0.940	0.914	0.031	0.40994	− 0.17	
21. Done things that overstepped my bounds	Impulsivity	0.927	0.916	0.031	0.70756	− 0.13	

^a^Benjamini-Hochberg rejects all p-values < 0.0016 to control the FDR at 0.05 and < 0.0003 to control the FDR at 0.01

^*^ < 5% false discovery rate (FDR), ** < 1% FDR

The scale ‘Existential values’ fitted the model (p = 0.846). The pairs of items 10 and 11, 10 and 13, and 12 and 13 had LD. Moreover, item 11 ‘Well-being’ possessed DIF related to the exogenous variable ‘Age’. Therefore, we tested the model without this item and with split for the variable ‘Age’. After we removed item 11, no DIF was revealed but overall fit to the model decreased (p = 0.032) as well as item fit of the remaining items in the scale. The pairs of items 2 and 10, 10 and 12, 10 and 13, and 12 and 13 had LD. We tested the model with split for the variable ‘Age’, and the item revealed non-uniform DIF i.e. overall fit decreased compared with the initial analyses (p = 0.750).

Both the scales and the items fitted the Rasch model in the initial analyses. Since item 10 and 11 possessed LD, we performed another analysis where we merged item 10 and 11 into a super item, to examine whether this would remove the DIF [20]. The merge of item 10 and 11 to a super item revealed an increased overall fit (p = 0.914) but did not remove the DIF and the fit of the super item was lower than that of item 11 in the previous analyses. Moreover, the items 10 and 13, and 12 and 13 also had LD why we one by one merged them into super items. None of these super items resulted in an increased item fit or removal of DIF. However, we did not delete item 11 from the model, due to its high content validity.

## Discussion

This study has developed and validated an extended version of COS specifically for use in CRC screening. The extended version is called consequences of screening in colorectal cancer, COS-CRC (Additional file 1). The extended version consists of three parts: part I (nine scales, two single items), part Ix (four scales and one single item) and part II (five scales).

The stringent design, combining qualitative and quantitative methods, is a strength of the study.

Moreover, all the items possessed high content validity and most of them also had adequate psychometric properties, which is a strength of this study.

Furthermore, COS has now proved content relevance and adequate measurement properties in five different screening settings, including CRC screening [19–23].

Only 16 persons of 80 invited men and women consented to participate in the focus groups, which could be considered a limitation [24]. However, since no new information developed during the last two group interviews, we were confident that data saturation was reached. Another limitation was the several scales that possessed LD. LD can decrease the item information collected and thereby the power of a study. However, presence of LD is not of importance as far as the scale fits the model and is used in a survey that has a sufficient number of respondents.

Moreover, the short recall period of four days could be considered another limitation. However, a longer recall period (e.g. a week) could induce inevitable bias, since it would not be possible to distinguish between consequences, in any directions, in relation to waiting for the iFOBT result, not having taken the iFOBT yet or even having undergone the colonoscopy.

The content relevance of the COS (part I and part II) as well as of the previously developed condition-specific items was established in a setting of CRC screening.

Furthermore, COS showed adequate measurement properties to measure psychosocial consequences in this context except in the scales ‘Introvert’, ‘Emotional reactions’, ‘Lifestyle changes’ and ‘Sexuality’ where one item in each scale possessed DIF. This may limit the applicability of these items to randomised studies, where DIF can be expected to be equally distributed among the study groups.

Item 41 in ‘Emotional reactions’ possessed uniform DIF related to the exogenous variable ‘Diagnosis’ but could be used in settings only investigating subgroups of CRC screening participants.

However, the study revealed gaps in content coverage of COS in relation to CRC screening-specific topics. New CRC screening-specific information was discovered in the focus groups and covered by 18 new items, which emphasize the importance of involving the experts when developing questionnaires. In this research area, the experts are the participants of the screening programme. COS-CRC is to our knowledge the first questionnaire on psychosocial consequences of CRC screening tested for content validity before use in CRC screening participants. The high content validity ensures that the questionnaire does not include items that are redundant or irrelevant to the respondents. Generic questionnaires are developed in other subpopulations than screening participants and have not been tested for content validity in a CRC screening setting [16, 17]. Therefore, there is a large risk that screening participants find these items irrelevant or redundant [39]. The high content validity of the COS-CRC questionnaire also confirms that all items in the questionnaire are relevant and are needed to cover all aspects of the multidimensional trait ‘Psychosocial consequences of CRC screening’ [39, 40].

Unexpectedly, the item ‘Well-being’ in the scale ‘Existential values’ possessed non-uniform DIF. This item has not possessed DIF in COS part II in screening for other non-communicable diseases [20–23]. As this DIF could be artificial, we therefore tried to locate the source of DIF by adding LD for three pairs of items to the model, thereby constructing super items [41]. This did not remove the DIF or increase the fit to the model. However, since this item has not possessed DIF in any other screening settings, the DIF could be spurious. Hence, this DIF should be tested in another sample before deleting this item permanently for use in a non-randomized CRC screening setting.

## Conclusion

An extended version of the questionnaire COS has been developed to measure psychosocial consequences of CRC screening. The measure is called consequences of screening in colorectal cancer (COS-CRC) and consists of three parts; Part I: ‘Anxiety’, ‘Behaviour’, ‘Dejection’, Sleep’, ‘Introvert’, ‘Fear and powerlessness’, ‘Change in body perception’, ‘Change in perception of own age’, ‘Emotional reactions’, and the two single items ‘Lifestyle changes’, and ‘Sexuality’; Part Ix: ‘Burden of bowel preparation’, Knowledge about colorectal polyps’, ‘Negative physical experiences of the colonoscopy’, ‘Negative emotional experiences of the colonoscopy’ and the single item on ‘Regret participation’ and Part II: ‘Relaxed/Calm’, ‘Social network’, Existential values’, ‘Impulsivity’, and ‘Empathy’. We showed using Rasch models, that COS-CRC possessed adequate measurement properties.

## Implications for research

We have not been able to identify any studies investigating the measurement properties of the questionnaires used to measure psychosocial consequences in a CRC setting, but in general, generic questionnaires are used for these purposes. However, condition-specific measures have been proved superior to generic measures in covering all the specific aspects of being part of a screening service [18]. Therefore, in future CRC screening trials measuring psychosocial consequences, condition-specific questionnaires with adequate measurement properties such as COS-CRC should be used to measure these consequences adequately. Moreover, suggestions for further research would be to include the two items on worries about CRC and believe in not having CRC in the COS-CRC to analyse whether these two items would fit a Rasch model.

## Supplementary Information

**Additional file 1.** COS-CRC questionnaire. An *ad hoc* English translation of the COS-CRC questionnaire.

## Data Availability

The datasets used and analysed during the current study are available from the corresponding author on reasonable request.

## Abbreviations

CRC: Colorectal cancer
iFOBT: Immunological feacal occult blood test
COS: Consequences of screening
LD: Local dependence
DIF: Differential item functioning
COS-CRC: Consequences of screening in colorectal cancer
