# Supplementary material for: Consequences of screening in colorectal cancer (COS-CRC): development and dimensionality of a questionnaire
Source: BMC Psychol. 2021 Jan 7;9:7. doi: 10.1186/s40359-020-00504-3 (PMC7792180; doi:10.1186/s40359-020-00504-3)
Supplement: Supplementary file 1 — Additional file 1. COS-CRC questionnaire. An ad hoc English translation of the COS-CRC questionnaire. [file 40359_2020_504_MOESM1_ESM.pdf]

Questionnaire

to people, who have participated in screening for colorectal cancer

With this questionnaire, we aim to collect information about how it affects people to participate in screening for colorectal cancer.

It is important that you respond to all the questions.

© Denmark: John Brodersen, The Research Unit for General Practice, The University of Copenhagen 2015

Part I

Have you, **during the last four days**, experienced any of the following?	Not at all	A bit	Quite a bit	A lot
1. I have been worried.	☐	☐	☐	☐
2. I have been worried about my future.	☐	☐	☐	☐
3. I have been scared.	☐	☐	☐	☐
4. I have been irritable.	☐	☐	☐	☐
5. I have been quieter than normal.	☐	☐	☐	☐
6. I have slept badly.	☐	☐	☐	☐

Have you, **during the last four days**, experienced any of the following?	Not at all	A bit	Quite a bit	A lot
7. I have had difficulties concentrating.	☐	☐	☐	☐
8. I have felt that time passed slowly.	☐	☐	☐	☐
9. My appetite has changed.	☐	☐	☐	☐
10. I have been sad.	☐	☐	☐	☐
11. I have been upset.	☐	☐	☐	☐
12. I have been restless.	☐	☐	☐	☐

Have you, **during the last four days**, experienced any of the following?	Not at all	A bit	Quite a bit	A lot
13. I have been nervous.	☐	☐	☐	☐
14. I have been uneasy.	☐	☐	☐	☐
15. It has taken a long time to fall asleep.	☐	☐	☐	☐
16. I have withdrawn into myself.	☐	☐	☐	☐
17. I have been unable to cope.	☐	☐	☐	☐
18. I have been depressed.	☐	☐	☐	☐

Have you, **during the last four days**, experienced any of the following?	Not at all	A few times	Some-times	Many times
19. I have had difficulties dealing with work or other commitments.	☐	☐	☐	☐
20. I have woken up far too early in the morning.	☐	☐	☐	☐
21. I have had difficulties doing things around the house.	☐	☐	☐	☐
22. I have been terrified.	☐	☐	☐	☐
23. I have been awake most of the night.	☐	☐	☐	☐

Have you, **during the last four days**, experienced any of the following?	Not at all	A bit	Quite a bit	A lot
24. I have felt sorry for myself.	☐	☐	☐	☐
25. I have been shocked.	☐	☐	☐	☐
26.* Fear of colorectal cancer has, more than ususal, been in the back of my mind.	☐	☐	☐	☐
27. I have felt insecure.	☐	☐	☐	☐
28. I have felt as though something was wrong with my body.	☐	☐	☐	☐

Have you, **during the last four days**, experienced any of the following?	Not at all	A bit	Quite a bit	A lot
29. I have felt as though my body was a machine that does not work.	☐	☐	☐	☐
30. I have thought my situation was hopeless.	☐	☐	☐	☐
31. I have experienced that I lost control.	☐	☐	☐	☐
32. I have experienced mood swings.	☐	☐	☐	☐

Have you, **during the last four days**, experienced any of the following?	Not at all	A bit	Quite a bit	A lot
33. I have thought my body was vulnerable.	☐	☐	☐	☐
34. I have kept my thoughts to myself.	☐	☐	☐	☐
35. I have felt that I am getting old.	☐	☐	☐	☐
36. I have felt sour (attitude).	☐	☐	☐	☐
37. I have been angry.	☐	☐	☐	☐
38. I have felt as I have been in a vacuum.	☐	☐	☐	☐

Have you, **during the last four days**, experienced any of the following?	Not at all	A bit	Quite a bit	A lot
39. I have felt older than my own age.	☐	☐	☐	☐
40. I have felt powerless.	☐	☐	☐	☐
41.* I have been frightened.	☐	☐	☐	☐
42. I have felt that I was unlucky.	☐	☐	☐	☐
43. I have changed diet.	☐	☐	☐	☐
44.* I have changed exercise habits.	☐	☐	☐	☐

Have you, **during the last four days**, experienced any of the following?	Not at all	A bit	Quite a bit	A lot	Do not know
45.* Less interest in sex.	☐	☐	☐	☐	☐
46. Negative impact on my sex life.	☐	☐	☐	☐	☐

Part Ix

Questions about the bowel preparation

Have you, **during the last four days**, experienced any of the following?	Not at all	A bit	Quite a bit	A lot
47. I felt discomfort with having the bowel emptied.	☐	☐	☐	☐
48. I have had a feeling of having a lesion on the rectum.	☐	☐	☐	☐
49. I have had a stinging feeling in the rectum.	☐	☐	☐	☐
50. I felt pain during emptying of bowel.	☐	☐	☐	☐
51. I have had a feeling of a rash on the backside.	☐	☐	☐	☐
52. I felt stranded at home during emptying of bowel.	☐	☐	☐	☐
53.* I was worried about drinking other fluids during emptying of bowel.	☐	☐	☐	☐

Questions about colorectal polyps

Have you, **during the last four days**, experienced any of the following?	Not at all	A bit	Quite a bit	A lot
54. Anxious about having one or more polyps.	☐	☐	☐	☐
55. I have been confused about what it means to have one or more polyps.	☐	☐	☐	☐

Questions about the colonoscopy

Have you, **during the last four days**, experienced any of the following?	Not at all	A bit	Quite a bit	A lot
56. I felt discomfort during the colonoscopy.	☐	☐	☐	☐
57. I felt defenseless lying on the examination table.	☐	☐	☐	☐
58. I felt vulnerable lying on the examination table.	☐	☐	☐	☐
59. I felt pain during the colonoscopy.	☐	☐	☐	☐
60. I felt humiliation lying on the examination table.	☐	☐	☐	☐
61. The examination was a harsh experience.	☐	☐	☐	☐
62. The examination overstepped my boundaries.	☐	☐	☐	☐
63. I felt exposed lying on the examination table.	☐	☐	☐	☐
64. I have had regrets having participated in the screening programme.	☐	☐	☐	☐

*Items deleted after the psychometric analyses

Part II

Considering everything, have the examinations resulted in any of the following:

Please choose only one alternative per question

---

1. After the examinations, my aspects of life have been:

☐ Much less broad	☐ Less broad	☐ **The same as before**	☐ More broad	☐ Much more broad
**Less broad aspects**			**More broad aspects**	

---

2. After the examinations, my enjoyment of life:

☐ Much less	☐ Less	☐ **The same as before**	☐ More	☐ Much more
**Less enjoyment**			**More enjoyment**	

---

3. After the examinations, I feel:

☐ Much less relaxed	☐ Less relaxed	☐ **As relaxed as before**	☐ More relaxed	☐ Much more relaxed
**Less relaxed**			**More relaxed**	

---

4. After the examinations, my abilities to listen to other people's problems:

☐ Much less	☐ Less	☐ **The same as before**	☐ More	☐ Much more
**Less listening**			**More listening**	

---

5. After the examinations, my understanding for other people's problems:

☐ Much less	☐ Less	☐ **The same as before**	☐ More	☐ Much more
**Less understanding**			**More understanding**	

Considering everything, have the examinations resulted in any of the following:

Please choose only one alternative per question

6. After the examinations, my relation to my family is:

☐ Much less close	☐ Less close	☐ **The same as before**	☐ More close	☐ Much more close
**Less close**			**More close**	

7. After the examinations, the relations to my friends are:

☐ Much less close	☐ Less close	☐ **The same as before**	☐ More close	☐ Much more close
**Mindre tæt**			**Mere tæt**	

8. After the examinations, my relations to other people are:

☐ Much worse	☐ Worse	☐ **The same as before**	☐ Better	☐ Much better
**Worse**			**Better**	

9. After the examinations, my feeling of inner calm is:

☐ Much less	☐ Less	☐ **The same as before**	☐ More	☐ Much more
**Less calm**			**More calm**	

10. After the examinations, my thoughts about the future are:

☐ Much less bright	☐ Less bright	☐ **The same as before**	☐ More bright	☐ Much more bright
**Less bright future**			**More bright future**	

11.* After the examinations, my feeling of well-being is:

☐ Much less	☐ Less	☐ **The same as before**	☐ More	☐ Much more
**Less well-being**			**More well-being**	

Considering everything, have the examinations resulted in any of the following:

Please choose only one alternative per question

---

12. After the examinations, my awareness of life is:

☐ Much less	☐ Less	☐ **The same as before**	☐ More	☐ Much more

Less awareness of life — **More awareness of life**

---

13. After the examinations I value life:

☐ Much less	☐ Less	☐ **The same as before**	☐ More	☐ Much more

Value life less — **Value life more**

---

14. After the examinations, I have:

☐ Much less energy	☐ Less energy	☐ **The same as before**	☐ More energy	☐ Much more Energy

Less energy — **More energy**

---

15. After the examinations, my feeling of responsibility for my family is:

☐ Much less	☐ Less	☐ **The same as before**	☐ More	☐ Much more

Less responsibility — **More responsibility**

---

16. After the examinations, I have lived life to the full:

☐ Much less	☐ Less	☐ **The same as before**	☐ More	☐ Much more

Live life less — **Live life more**

Considering everything, have the examinations resulted in any of the following:

Please choose only one alternative per question

17. After the examinations, I feel:

☐ Much less relieved	☐ Less relieved	☐ **The same as before**	☐ More relieved	☐ Much more relieved
**Less relieved**			**More relieved**	

18. After the examinations, I am:

☐ Much less impulsive	☐ Less impulsive	☐ **The same as before**	☐ More impulsive	☐ Much more impulsive
**Less impulsive**			**More impulsive**	

19. After the examinations, my desire to venture into something new is:

☐ Much weaker	☐ Weaker	☐ **The same as before**	☐ Stronger	☐ Much stronger
**Weak desire**			**Stronger desire**	

20. After the examinations, my courage to venture into something risky is:

☐ Much weaker	☐ Weaker	☐ **The same as before**	☐ Stronger	☐ Much stronger
**Less courage**			**Mere modig**	

21. After the examinations, I have done things that overstepped my bounds:

☐ Much less	☐ Less	☐ **The same as before**	☐ More	☐ Much more
**Less**			**More**	

*Deleted after psychometric analyses
